# Dataset on specifications, carcinogenic and non-carcinogenic risk of volatile organic compounds during recycling paper and cardboard

**DOI:** 10.1016/j.dib.2020.105296

**Published:** 2020-02-17

**Authors:** Ramin Nabizadeh, Armin Sorooshian, Mahdieh Delikhoon, Abbas Norouzian Baghani, Somayeh Golbaz, Mina Aghaei

**Affiliations:** a Department of Environmental Health Engineering, School of Public Health, Tehran University of Medical Sciences, Tehran, Iran; b Center for Air Pollution Research (CAPR), Institute for Environmental Research (IER), Tehran University of Medical Sciences, Tehran, Iran; c Department of Chemical and Environmental Engineering, University of Arizona, Tucson, AZ, USA; d Department of Hydrology and Atmospheric Sciences, University of Arizona, Tucson, AZ, USA; e Department of Occupational Health Engineering, School of Public Health, Isfahan University of Medical Sciences, Isfahan, Iran

**Keywords:** Paper and cardboard recycling, VOCs, Inhalation lifetime cancer risk, Exposure indices, Hazard quotient

## Abstract

Emissions of volatile organic compounds (VOCs) were studied during paper and cardboard recycling from a paper and cardboard solid waste recycling factory (PCSWRF). Data are summarized in this article for the following quantities for a PCSWRF during the winter in Tehran, Iran: VOC concentrations (μg m^−3^), the percentage of detected VOCs, exposure indices (E_i_) of individual and total VOCs (TVOCs), inhalation lifetime cancer risk (LTCR) of VOCs, the hazard quotient (HQ) of VOCs, sensitivity analysis (SA) for VOC exposure in different age groups (birth to <81), and Spearman's rank correlation coefficients (r) between VOC concentrations and meteorological parameters. For more insight please see “Characteristics and Health Effects of Volatile Organic Compound Emissions during Paper and Cardboard Recycling”[1], https://doi.org/10.1016/j.scs.2019.102005.

**Table:** Specifications Table

Subject	Environmental Science
Specific subject area	Environmental air pollution and Health
Type of data	Table and Figure
How data were acquired	Active sampling (Low Flow Sample Pump 222 Series, SKC Inc.), GC-MS (GC 7890N, AGILENT- MS 5975C, MODE EI.MS)
Data format	Analyzed
Parameters for data collection	Sampling, extraction and analysis parameters are briefly described in this paper and fully provided in the related research article.
Description of data collection	Data were collected using active sampling (SKC 222 Series Low Flow Pump) with a charcoal glass tube and using gas chromatography–mass spectrometry (GC-MS) (GC 7890N, AGILENT- MS 5975C, MODE EI.MS). An HP- 5MS column (60 m × 0.32 mm × 0.25 μm, Agilent Technologies,USA) was used.
Data source location	Descriptive data were obtained in a paper and cardboard solid waste recycling factory (PCSWRF) located in Tehran, Iran. Latitude: 35°32′42"N, longitude: 51°23′35"E.
Data accessibility	Repository name: Mendeley Data Data identification number: https://doi.org/10.17632/jmtkhgxp9v and https://doi.org/10.1016/j.scs.2019.102005
Related research article	R. Nabizadeh, A. Sorooshian, M. Delikhoon, A. N. Baghani1, S. Golbaz, M. Aghaei, Characteristics and Health Effects of Volatile Organic Compound Emissions during Paper and Cardboard Recycling. Sustainable Cities and Society (SCS) (2019) [1], https://doi.org/10.1016/j.scs.2019.102005

**Table:** 

**Value of the data** • The data could be used by researchers to further investigate risk assessment of workers' exposure to volatile organic compounds (VOCs) during paper and cardboard recycling in different regions. • The data could be applied by researchers to study photochemical aging and to find emission sources of VOCs. • The data provides valuable information on the relationships between VOC concentrations and meteorological parameters. • The data allows comparison between the concentration of VOC species in different areas of paper and cardboard solid waste recycling factory (PCSWRF).

## 1 Data description

**Fig. 1:**
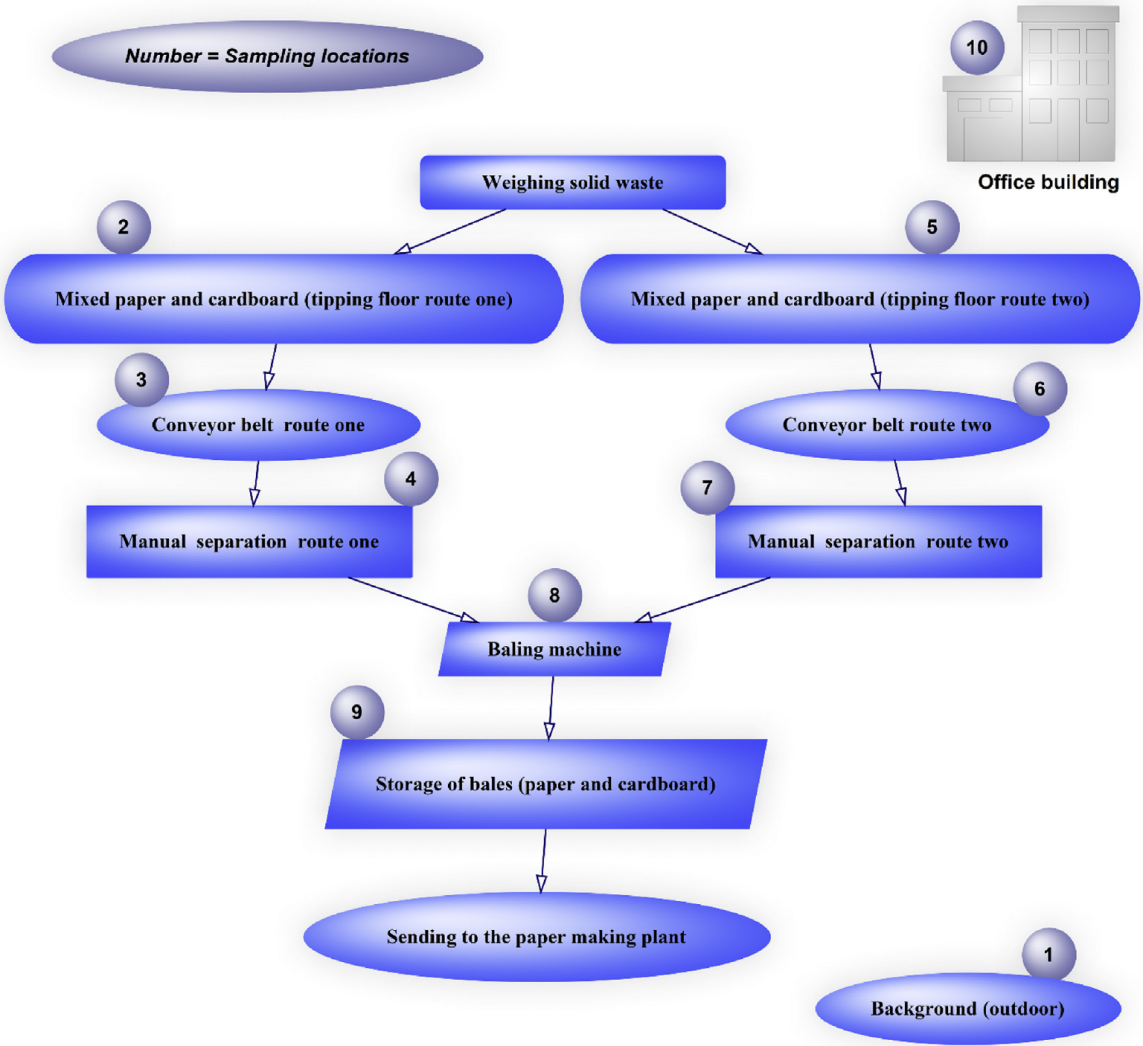
A diagram of sampling points in the PCSWRF.

**Fig. 2:**
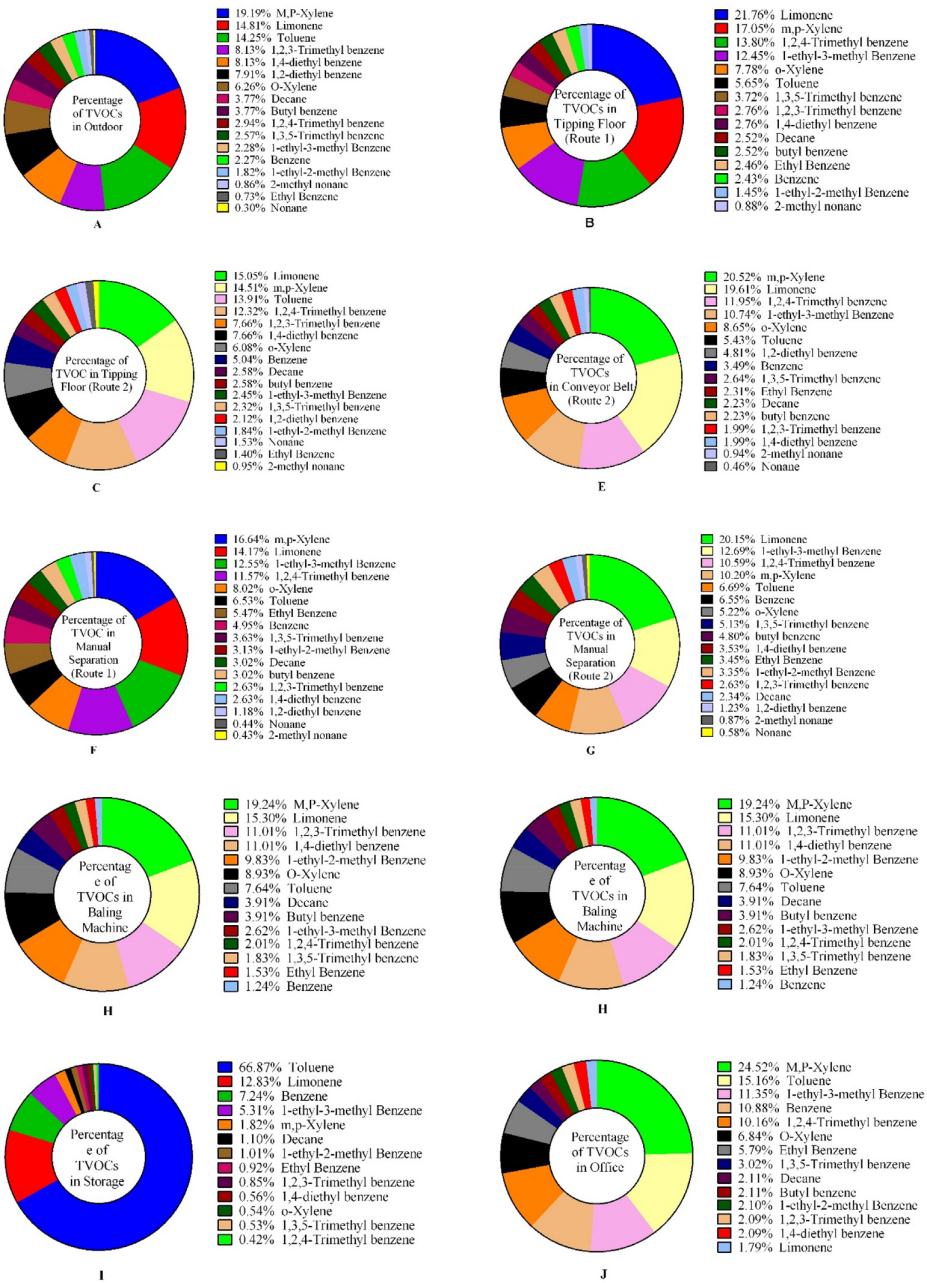
The percentage of detected VOCs based on frequency in different sampling sites: background (A); tipping floor route/line one (B); tipping floor route/line two (C); conveyor belt line one (D); conveyor belt line two (E); manual separation line one (F); manual separation line two (G); baling machine (H); storage (I); office (J).

**Fig. 3:**
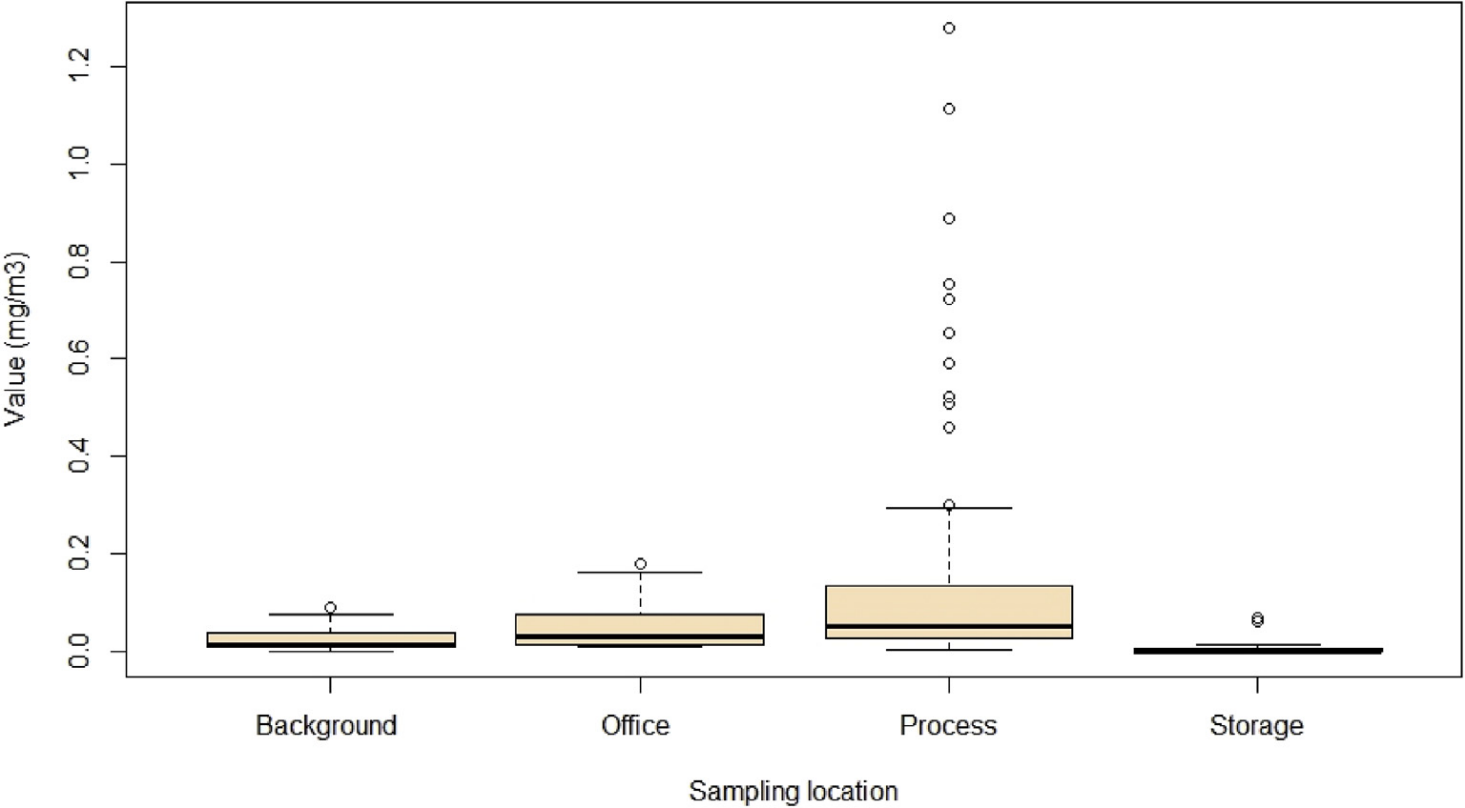
Box plot of VOC concentrations in different sampling locations in winter.

**Fig. 4:**
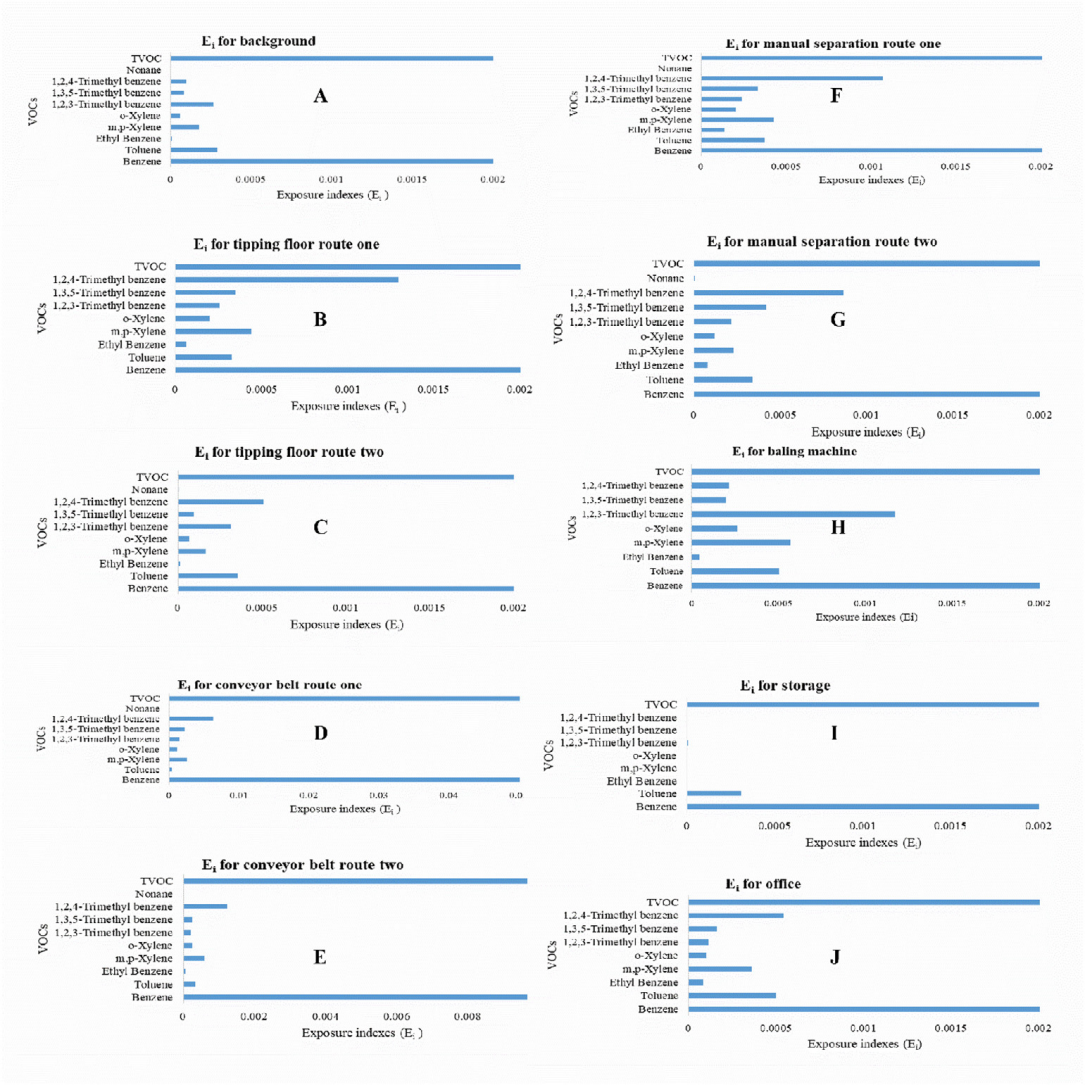
Exposure indices (E_i_) of individual and TVOCs in different sites of the PCSWRF: background (A); tipping floor route/line one (B); tipping floor route/line two (C); conveyor belt line one (D); conveyor belt line two (E); manual separation line one (F); manual separation line two (G); baling machine (H); storage (I); office (J).

**Fig. 5:**
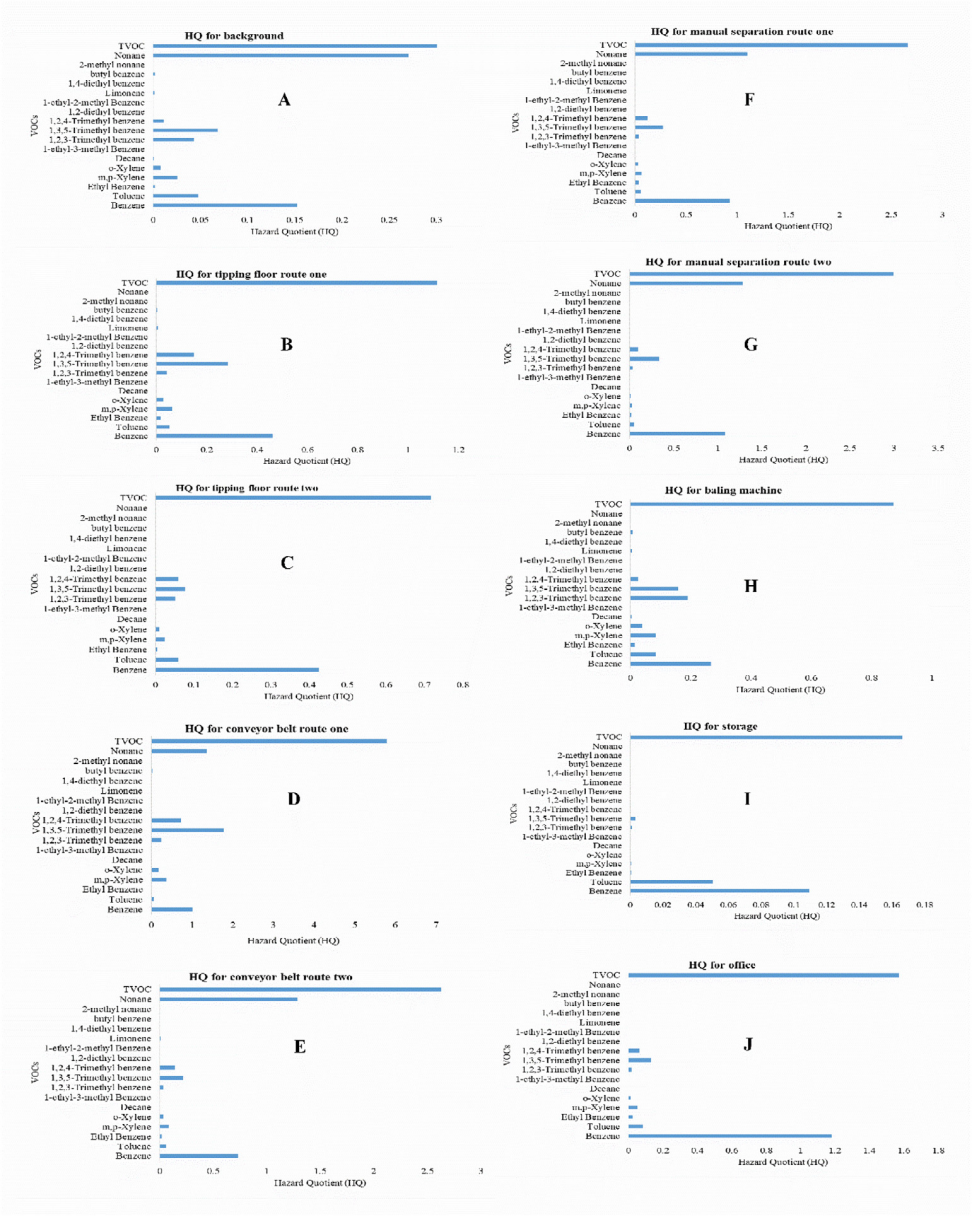
The hazard quotient (HQ) of individual and TVOCs in different sites from PCSWRF: background (A); tipping floor route/line one (B); tipping floor route/line two (C); conveyor belt line one (D); conveyor belt line two (E); manual separation line one (F); manual separation line two (G); baling machine (H); storage (I); office (J).

**Fig. 6:**
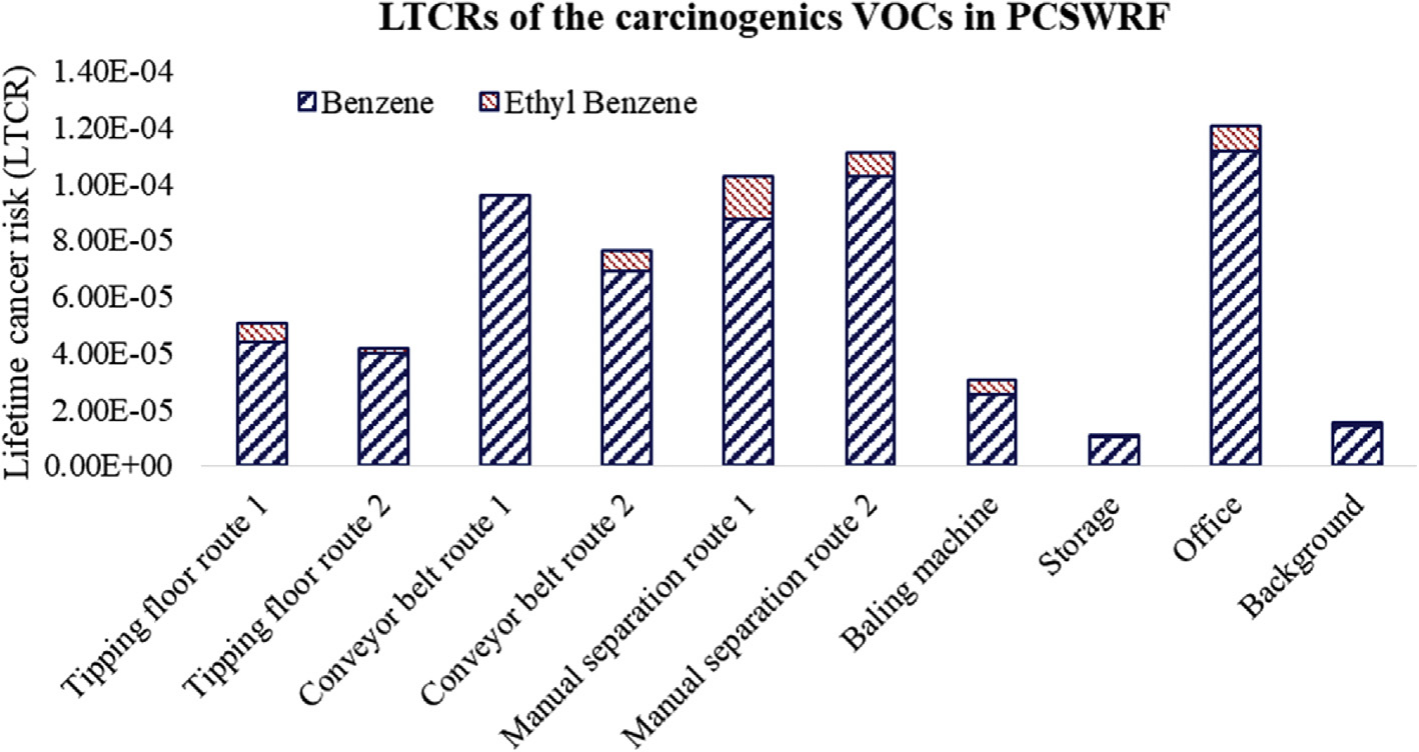
The LTCRs of carcinogenic VOCs in the PCSWRF.

We collected data on VOCs species using GC-MS for different areas of a paper and cardboard solid waste recycling factory (PCSWRF) in different meteorological conditions. The six tables and two figures that are provided as data for this article contain a diagram of sampling points (Fig. 1), the percentage and box plot of VOCs (Fig. 2, Fig. 3), exposure indices (Ei) (Fig. 4) and hazard quotient (HQ) of individual and TVOCs (Fig. 5), inhalation lifetime cancer risk (LTCR) of VOCs (Fig. 6), The threshold limit value-time-weighted average (TLV-TWA), the reference dose (RfD), and cancer slope factor (CSF) of VOCs (Table 1), and also Pearson's correlation between VOC concentrations and meteorological parameters (Table 2).

**Table 1:** TLV-TWA, RfD, CSF, and their carcinogenic classifications in IARC of recognized VOCs.

VOCs	TLV-TWA^a^ (mg/m^3^)	RfD^b^ (mg kg^−1^ day^−1^)	Source	CSF^c^ (mg^−1^ kg day)	Group IARC^d^
Nonane	1.1 × 10^3^	0.0003	PPRTV^e^	–	–
Decane	–	1	PPRTV	–	–
Benzene	1.7 × 10^−3^	0.0040 (Oral)^g^	IRIS^f^	0.055	1
Toluene	2.1 × 10^2^	0.080	IRIS	–	–
Ethylbenzene	4.7 × 10^2^	0.10	IRIS	0.0087	2B
M,P-Xylene	4.7 × 10^2^	0.20	IRIS	–	–
O-Xylene	4.7 × 10^2^	0.20	IRIS	–	–
1,3,5-Trimethylbenzene	1.3 × 10^2^	0.010	PPRTV	–	–
1,2,4-Trimethylbenzene	1.3 × 10^2^	0.070	PPRTV	–	–
1,2,3-Trimethylbenzene	1.3 × 10^2^	0.050	PPRTV	–	–
1,2-diethyl benzene	–	–	–	–	–
1-ethyl-2-methyl Benzene	–	–	–	–	–
Limonene		2.5^h^	(1)^h^		–
1,4-diethyl benzene= appendix	–	0.1		0.0110 Oral	
Butyl benzene	–	0.10 Subchronic^i^	IRIS	–	–
2-methyl nonane	–	–	–	–	–
1-ethyl-3-methyl Benzene	–	–	–	–	–

a TLV-TWA: Data provided by ACGIH.

b RfD: Reference dose for chronic oral exposure; CSF: Cancer Slope factor (CSF obtained from IRIS).

d IARC: International Agency for Research on Cancer.

e PPRTV: Provisional Peer Reviewed Toxicity Values of IRIS.

f IRIS: Integrated Risk Information system.

g Human occupational inhalation study: Rothman,1996.

h [10].

i (0.50 Chronic p-RfD).

**Table 2:** Pearson's correlation between VOC concentrations based on average concentrations for all sites. Relationships between VOC concentrations and meteorological parameters are shown too.

Components		X1	X2	X3	X4	X5	X6	X7	X8	X9	X10	X11	X12	X13	X14	X15	X16	X17	X18	X19

X1	r P-value	1	.422 .224	.807^b^ .009	.397 .257	.378 .282	.347 .326	.511 .131	.068 .853	.448 .194	.456 .185	.268 .454	.212 .557	.351 .320	.088 .809	.413 .235	.385 .272	.409 .240	-.450 .192	.194 .592
X2	r P-value	.422 .224	1	.334 .380	.435 .209	.408 .242	.432 .213	.299 .402	.594 .070	.319 .369	.286 .424	-.013 .971	.618 .057	.209 .562	.589 .073	.405 .245	-.240 .505	-.140 .699	.375 .285	-.351 .321
X3	r P-value	.807^b^ .009	.334 .380	1	.565 .113	.566 .112	.472 .199	.820^b^ .007	-.053 .892	.758^a^ .018	.693^a^ .039	.001 .998	.148 .705	.409 .274	-.038 .922	.506 .164	.274 .475	.208 .591	-.343 .367	.106 .785
X4	r P-value	.397 .257	.435 .209	.565 .113	1	.998^b^ .000	.988^b^ .000	.967^b^ .000	.809^b^ .005	.975^b^ .000	.973^b^ .000	.742^a^ .014	.852^b^ .002	.843^b^ .002	.807^b^ .005	.968^b^ .000	.562 .091	.315 .376	-.353 .316	-.113 .755
X5	r P-value	.378 .282	.408 .242	.566 .112	.998^b^ .000	1	.994^b^ .000	.970^b^ .000	.819^b^ .004	.980^b^ .000	.975^b^ .000	.735^a^ .016	.863^b^ .001	.862^b^ .001	.818^b^ .004	.977^b^ .000	.575 .082	.333 .347	-.381 .277	-.079 .828
X6	r P-value	.347 .326	.432 .213	.472 .199	.988^b^ .000	.994^b^ .000	1	.950^b^ .000	.865^b^ .001	.973^b^ .000	.955^b^ .000	.694^a^ .026	.907^b^ .000	.853^b^ .002	.865^b^ .001	.987^b^ .000	.525 .119	.312 .379	-.370 .292	-.044 .905
X7	r P-value	.511 .131	.299 .402	.820^b^ .007	.967^b^ .000	.970^b^ .000	.950^b^ .000	1	.673^a^ .033	.989^b^ .000	.994^b^ .000	.731^a^ .016	.755^a^ .012	.857^b^ .002	.676^a^ .032	.952^b^ .000	.669^a^ .034	.386 .271	-.547 .102	.024 .947
X8	r P-value	.068 .853	.594 .070	-.053 .892	.809^b^ .005	.819^b^ .004	.865^b^ .001	.673^a^ .033	1	.738^a^ .015	.687^a^ .028	.450 .192	.971^b^ .000	.717^a^ .020	.999^b^ .000	.844^b^ .002	.229 .524	.134 .711	.009 .981	-.181 .616
X9	r P-value	.448 .194	.319 .369	.758^a^ .018	.975^b^ .000	.980^b^ .000	.973^b^ .000	.989^b^ .000	.738^a^ .015	1	.990^b^ .000	.715^a^ .020	.812^b^ .004	.838^b^ .002	.743^a^ .014	.977^b^ .000	.612 .060	.380 .279	-.529 .116	.061 .866
X10	r P-value	.456 .185	.286 .424	.693^a^ .039	.973^b^ .000	.975^b^ .000	.955^b^ .000	.994^b^ .000	.687^a^ .028	.990^b^ .000	1	.747^a^ .013	.752^a^ .012	.843^b^ .002	.688^a^ .028	.948^b^ .000	.666^a^ .036	.413 .236	-.531 .114	.000 1.000
X11	r P-value	.268 .454	-.013 .971	.001 .998	.742^a^ .014	.735^a^ .016	.694^a^ .026	.731^a^ .016	.450 .192	.715^a^ .020	.747^a^ .013	1	.430 .215	.679^a^ .031	.448 .194	.680^a^ .030	.710^a^ .022	.549 .100	-.361 .305	-.031 .932
X12	r P-value	.212 .557	.618 .057	.148 .705	.852^b^ .002	.863^b^ .001	.907^b^ .000	.755^a^ .012	.971^b^ .000	.812^b^ .004	.752^a^ .012	.430 .215	1	.732^a^ .016	.974^b^ .000	.902^b^ .000	.230 .522	.130 .720	-.123 .736	-.051 .889
X13	r P-value	.351 .320	.209 .562	.409 .274	.843^b^ .002	.862^b^ .001	.853^b^ .002	.857^b^ .002	.717^a^ .020	.838^b^ .002	.843^b^ .002	.679^a^ .031	.732^a^ .016	1	.726^a^ .017	.872^b^ .001	.809^b^ .005	.401 .251	-.383 .274	-.139 .702

X1:Benzene; X2: Toluene; X3: Ethyl Benzene; X4: M,P-Xylene; X5: O-Xylene; X6: Decane; X7: 1-ethyl-3-methyl Benzene; X8: 1,2,3-Trimethyl benzene; X9: 1,3,5-Trimethyl benzene; X10: 1,2,4-Trimethyl benzene; X11: 1,2-diethyl benzene; X12: 1-ethyl-2-methyl Benzene; X13: Limonene; X14: 1,4-diethyl benzene; X15: Butyl benzene; X16: 2-methyl nonane; X17: Nonane; X18: Temperature; X19: Humidity.

a Correlation is significant at the 0.05 level (2-tailed).

b Correlation is significant at the 0.01 level (2-tailed).

## 2 Experimental design, materials, and methods

### 2.1 Study area

The capital of Iran is Tehran (35°32′42"N, 51°23′35"E) with around 13.31 million inhabitants according to a census report [2]. Measurements were specifically conducted at a PCSWRF. This factory has two lines of separation processes for paper and cardboard, including a tipping floor (line one and two), conveyor belt (line one and two), hand picking/manual separation (line one and two), and finally a baling machine (Fig. 1) (see Fig. 2, Fig. 3, Fig. 4, Fig. 5, Fig. 6).

About 3000 kg/day solid waste are transferred to this factory on a daily basis, comprised of paper and cardboard (more than 90%) and some other waste (lower than 10%) containing organic wastes, glass, aluminum, plastics, textiles, metals, leather, and wood. To date, 102 workers (88 in operational units and 14 in offices) have worked in this factory, which is 16000 m^2^ in area. In this factory, the weight of each package (bale) ranges between 1000 kg and 1700 kg, and bales are stored in the storage site. Most workers do not use personal protective equipment (PPE), including respirators or gloves.

### 2.2 Sampling and analysis

Sampling was carried out based on the U.S.EPA TO-15 method [1,3] and conducted over 2 h from 22 December 2017 to 20 February 2018 by active sampling (Low Flow Sample Pump 222 Series, SKC Inc.) with charcoal sorbent tubes (SKC Inc.) at a flow rate of 0.2 L min^−1^ [4,5]. Sampling was done at a height of 2 m in the PCSWRF. Before analysis, two charcoal beds in each tube (the back and front) were set into separate vials and the target pollutants were elicited by adding one ml CS_2_ [3]. Target pollutants were tested by GC-MS (GC 7890N, AGILENT- MS 5975C, MODE EI.MS). For the 10 sampling sites (Fig. 1), a total of 100 VOC samples were collected between December and February.

### 2.3 Statistical analysis

SPSS analytical software (Version 22.00) was used for statistical analysis. The Fligner-Killeen test was applied to check for homogeneity of variance. If the p-value obtained from the Fligner-Killeen test exceeded 0.05, the ANOVA test was performed for further analysis. But, if the p-value was less than 0.05, the Kruskal-Wallis test was applied for further analysis. Finally, if the Kruskal–Wallis test was significant, the Kruskal-Wallis post-hoc test (Kruskal Mac) was carried out to show that levels of the independent variable vary from other levels.

### 2.4 Health risk assessment for VOCs

For calculating inhalation lifetime cancer risk (LTCR) for VOC compounds, Eq. (1) was used, while Eq. (2) was applied to assess the non-carcinogenic risk or hazard quotient (HQ) for VOC compounds [[4], [5], [6], [7], [8]].

(1) LTCR = ((C × IR × ED × EF)/(AT × BW))× CSF

(2) HQ = ((C × IR × ED × EF)/(AT × BW))/RfD where; (Unsafe) 1< HQ≤1 (Safe)

C and IR represent pollutant concentrations (μg/m^3^) and human inhalation rate (m^3^ day^−1^), respectively. ED and EF represent the exposure duration (year) and exposure frequency (days year^−1^), respectively. BW and AT are the body weight (kg) and the average lifetime (days), respectively. HQ, RfD and CSF are hazard quotient (mg kg^−1^ day^−1^), reference dose (mg kg^−1^ day^−1^) and cancer slope factor (mg kg^−1^ day^−1^)^−1^), respectively [9].
